# Hollow Channel Formation inside Sodium Aluminoborate Glass by Femtosecond Laser Writing and Distilled Water Etching

**DOI:** 10.3390/ma14195495

**Published:** 2021-09-23

**Authors:** Sergey Fedotov, Alexey Lipatiev, Tatiana Lipateva, Sergey Lotarev, Vladimir Sigaev

**Affiliations:** Department of Chemical Technology of Glass and Glass Ceramics, Mendeleev University of Chemical Technology, 125480 Moscow, Russia; lipatievas@yandex.ru (A.L.); t.lipateva@yandex.ru (T.L.); sergey_lot@mail.ru (S.L.); vlad.sigaev@gmail.com (V.S.)

**Keywords:** femtosecond laser direct writing, nanograting, polarization-sensitive birefringence, sodium aluminoborate glass, selective etching

## Abstract

Recently, the effect of nanograting formation was demonstrated for binary sodium borate glass with the possibility of data storage with an enhanced level of security. The obvious disadvantage of such glass is poor chemical stability, which limits real applications. In this paper, we show that the introduction of Al_2_O_3_ allows preserving the possibility of nanograting formation with a significant increase of chemical resistance and thus to preserve optical memory applications. On the other hand, the possibility of selective etching of laser-written tracks by means of distilled water is revealed, which was not demonstrated for other types of glasses. The dependence of retardance of nanogratings form birefringence on laser writing parameters is established and discussed. Structural features of laser-modified microdomains are studied via Raman spectroscopy which revealed an increase of three-coordinated boron content. A possible mechanism of selective etching is discussed.

## 1. Introduction

Femtosecond laser-assisted etching (FLAE) is actively used for creation of different micro- [1], opto- [2] and electrofluidic devices [3]. FLAE is based on the writing of the desired architecture using femtosecond laser pulses in the bulk of the material and subsequent selective etching of the laser-modified areas. The key parameters of the etching process are the etching rate and selectivity which depend on the host material as well as on the etching reagent. For example, the most effective etching of Nd:YAG single crystal with high selectivity was achieved using orthophosphoric acid at 77 °C [4]. The most common material among glasses serving for the development of microfluidic devices is silica glass due to its unique optical and thermal properties and excellent chemical resistance. Generally, two etching agents namely hydrofluoric acid (HF) and potassium hydroxide (KOH) are used for silica glass chemical processing. A direct comparison of these etching reagents was made by Ross et al. [5] who showed that the etching rate of 5% HF at 40 °C was 320 μm/h, while KOH at 80 °C provided an etching rate of about 363 μm/h. Importantly, it was confirmed that the selectivity of the etching process, expressed by the ratio of the length of the etched channel to the change of the sample size, in the case of KOH is 1.5 orders of magnitude higher than for HF: up to 1000 and 70, respectively. Besides the choice of the etching agent, the laser writing conditions of the track also play an important role. Thus, the formation of nanogratings with nanoplanes elongated along a track is known to promote a higher rate of track etching in comparison with other types of silica glass modifications [6]. The possibility of selective etching of laser-written tracks in various glasses beyond SiO_2_ is now being intensively investigated and to date was shown for aluminoborosilicate [7], borosilicate [8], and BK7 [9] glasses.

Recently, we have demonstrated the laser-induced formation of birefringent nanogratings in the bulk of sodium borate glasses [10]. Such glasses are well known for their hygroscopicity [11]. However, the introduction of aluminum oxide can significantly improve their chemical durability [12,13]. Thus, sodium aluminoborate glasses are promising candidates for the implementation of the FLAE technique. In this letter, we demonstrate the formation of nanogratings in the volume of sodium aluminoborate glass, as well as the laser inscription of continuous tracks which can be etched at rates higher than those obtained for previously studied glasses [5].

## 2. Materials and Methods

Sodium aluminoborate glass was fabricated using the melt-quenching technique. High pure H_3_BO_3_ (99.9 wt%), Na_2_CO_3_ (99.8 wt%), Al_2_O_3_ (99.9 wt%) were used as components for glass batch preparation. The thoroughly mixed reagents were melted for 1 h in a platinum crucible in an electric furnace with silicon carbide heaters at a temperature of 1200 °C. The crucible was covered with a platinum lid to reduce the volatilization of the components. Glass was fabricated by casting the melt on a steel plate and subsequent pressing with another steel plate. Then as-quenched glass was annealed at 450 °C. The actual chemical composition of glass was determined by inductively coupled plasma mass spectrometry (XSeriesII ICP-MS, Thermo Scientific Inc., Waltham, MA, USA) for which the glass samples were preliminarily dissolved in nitric acid. The density measurement was carried out by hydrostatic weighing. Glass transition temperature T_g_ was determined by differential scanning calorimetry (Netzch STA 449F3), the heating rate was 10 °C/min. The refractive index was determined using Abbe refractometer Atago NAR-3T.

A regeneratively amplified femtosecond laser Pharos SP (Light Conversion Ltd., Vilnius, Lithuania) operating at 1030 nm was used for the micromodification of the glass sample. A laser beam was focused at depth of 50 µm under the plane surface of the sample using Olympus 20× microscope objective lens (N.A. = 0.45). Pulse duration, repetition rate, pulse energy and number of pulses were varied from 180 fs to 2000 fs, from 10 kHz to 1 MHz, from 20 to 400 nJ, from 10 to 10^7^ pulses, respectively. A glass sample was mounted on the computer-controlled Aerotech ABL1000 air-bearing 3D translation stage which provided high-precision positioning and movement of the sample relative to the laser beam.

Laser-modified areas were examined using optical microscopy (Olympus BX-51), quantitative microanalysis of birefringence (Abrio Microbirefringence, CRi Inc. Chantilly, VA, USA) at the wavelength of 546 nm, confocal Raman spectroscopy with the excitation by Ar ion laser at the wavelength of 488 nm and atomic force microscopy (AFM) using Ntegra Spectra facility (NT-MDT Ltd., Moscow, Russia) in the contact mode. The Raman spectra were registered by a cooled CCD camera with a resolution of 1 cm^−1^. Chemical etching was performed by dipping glass samples with laser-written tracks in the container with the etching agent at room temperature.

## 3. Results and Discussion

The results of inductively coupled plasma mass spectroscopy performed for synthesized glass show that the actual content of 31 mol.% Na_2_O, 8 mol.% Al_2_O_3_, 61 mol.% B_2_O_3_ is satisfactorily close to the nominal glass composition 35Na_2_O·10Al_2_O_3_·55B_2_O_3_ (mol%). The glass transition temperature T_g_, the glass density and the refractive index were measured as 464 ± 2 °C, 2.32 ± 0.01 g/cm^3^, and 1.492 ± 0.001 respectively and are in good coincidence with previously reported data for the similar glass composition.

**Figure 1:**
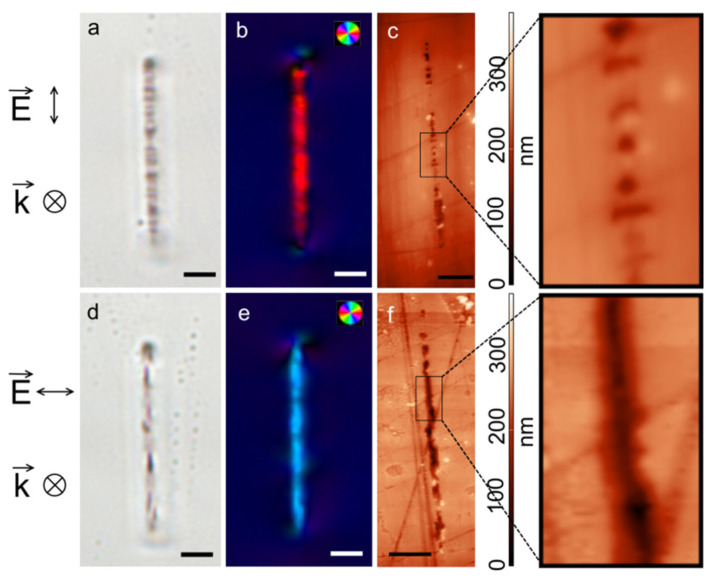
(**a**,**d**) Optical microphotographs; (**b**,**e**) pseudocolor maps of the slow axis of birefringence; (**c**,**f**) AFM images of the tracks inscribed by the laser beam with the polarization parallel (top) and perpendicular (bottom) to the track orientation, respectively (writing conditions: 10 kHz, 3 μm/s, 250 nJ, 600 fs). E—electromagnetic field vector, k—wave vector of the laser beam. The scale bars are 5 µm.

The preliminary study of the laser-modified microregions using a system for quantitative analysis of birefringence showed that it is possible to obtain polarization-dependent birefringence with a slow axis perpendicular to laser writing beam polarization in the bulk of sodium aluminoborate glass by direct laser writing. By analogy with data on laser writing in sodium borate glasses [10] one can conclude that observed birefringence in laser-inscribed dots is associated with nanogratings formation and their inherent form birefringence. Figure 1 shows the results of optical microscopy and atomic force microscopy carried out for the tracks written at two mutually perpendicular laser beam polarizations at laser exposure conditions providing nanogratings appearance. Optical brightfield micrographs allow to underline differences in the morphology of tracks that is transverse inhomogeneities are visible in a track written with the direction of polarization along the scanning direction while the orientation of the inhomogeneities become to coincide with the track in the case of the laser polarization perpendicular to the laser writing direction. Microbirefringence analysis (Figure 1b,e) of tracks shows that as well as for laser-written dots the direction of the slow axis is perpendicular to the direction of laser beam polarization. Finally, the AFM study of laser-inscribed tracks exposed by optical-grade mechanical polishing and subsequently etched in 0.12 M KOH aqueous solution during several seconds confirms their nanograting structure, which is especially clearly observed for a track, written with the polarization directed along the scanning direction. The retardance provided by form birefringence of the presented tracks is 35–40 nm, and the nanoplanes period is about 0.5–0.6 µm.

**Figure 2:**
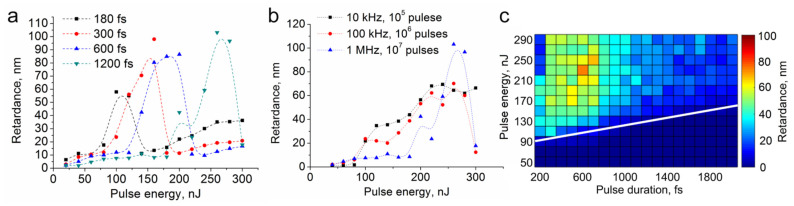
(**a**) Dependence of retardance on the pulse energy in the dots written at 1 MHz repetition rate; (**b**) Dependence of retardance on pulse energy in the dots written at 600 fs pulse duration; (**c**) Dependence of retardance on the pulse energy and pulse duration in the tracks written at 10 kHz repetition rate and 3 µm/s scanning speed.

Then we studied the influence of the laser exposure parameters on the formation of birefringent nanogratings in more detail. It turned out that, like in the case of sodium borate glass [10], a low pulse repetition rate and long duration are favorable for the formation of nanogratings. For example, at the lowest available pulse duration of 180 fs, nanogratings could be formed only at a repetition rate of 1 MHz in a narrow pulse energy range of 100–120 nJ. Within an increase of the pulse duration the pulse energy range of nanogratings formation shifts towards higher values (Figure 2a). A decrease in the repetition rate leads, firstly, to an expanding of the pulse energy range and, secondly, to a decrease in the minimum number of pulses required for the form birefringence appearance (Figure 2b), i.e., from 10^7^ pulses for 1 MHz, to 10^4^ in the case of 10 kHz.

A set of lines was written at a pulse repetition rate of 10 kHz and a scanning speed of 3 μm/s, which corresponds to an overlap of 10^4^ pulses per spot, in order to determine the dependence of the retardance on the pulse energy and duration. A two-dimensional plot of this dependence is shown in Figure 2c. It can be seen that an increase of the pulse energy leads to the growth of retardance, while the dependence of retardance on the pulse duration has an extremal character with a maximum in the range of 600–700 fs. The white line marks the bottom boundary of the range enabling the formation of nanogratings. Similar to the data reported earlier [14], the threshold of the formation of nanogratings depends on the laser intensity. Nevertheless, the estimation of this threshold gives a value of about 2.1 TW/cm^2^ for aluminoborate glass under study that is twice higher than one for silica or borosilicate glasses. It is known that nanogratings consist of nanoplanes with defects and nanopores. The necessary condition for their formation is the breaking of bonds in the glass network. However, the difference in the strength of B-O and Si-O bonds is not substantial [15]. So, other factors such as cation migration [16] or defects trapping [17] should be taken into account.

**Figure 3:**
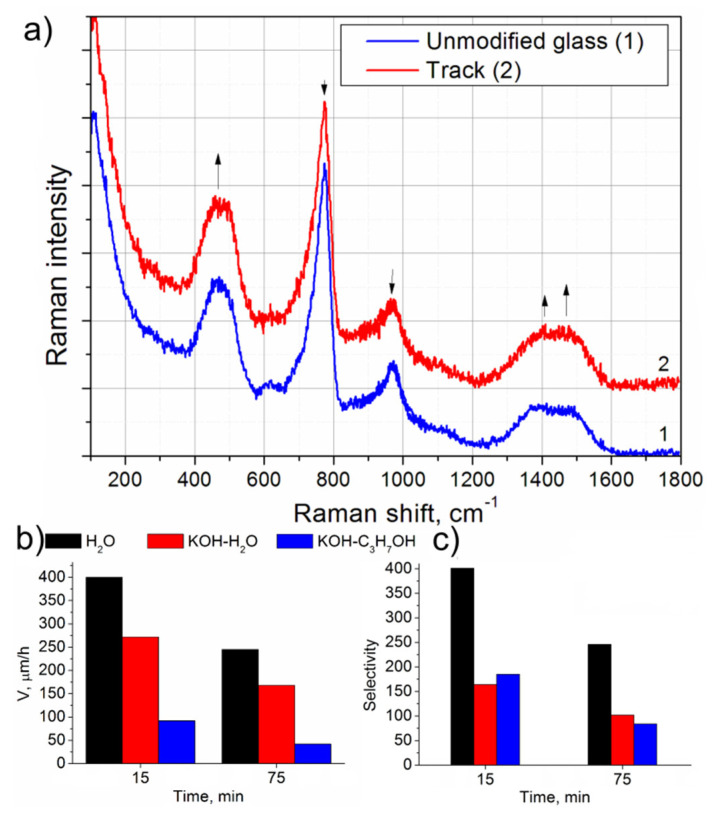
(**a**) Raman spectra of pristine glass and the laser-written track; (**b**) Etching rate of the laser-written tracks, (**c**) etching selectivity.

The crosscuts of the laser-inscribed tracks and initial glass were studied using Raman spectroscopy to understand structural rearrangements in glass after laser irradiation. Figure 3a shows Raman spectra of unmodified glass and the laser-written track. The spectrum of pristine glass exhibits peaks with maxima located at 460, 772, 965, 1120, and 1410 cm^−1^ characteristic for sodium borate and aluminoborate glass [10,18]. They correspond, respectively, to vibrations of isolated diborate groups, B-O-B bending mode and symmetric breathing vibration of six-membered rings with BO_4_ tetrahedron, vibrations of orthoborate groups, and stretching vibrations of [BO_3_] groups with 3-coordinated boron atoms [18,19]. The spectra of the modified region are characterized by the presence of the same peaks, however, with a different relative intensity. A decrease in the relative intensity of the peaks near 772, and 965 cm^−1^, as well as its increase for the peaks at 460 and 1410 cm^−1^ are observed in the spectrum of the modified region. A slight redistribution of intensities of the bands composing the complex peak at 1300–1600 cm^−1^ expressed in the growth of a high-frequency shoulder at ~1480 cm^−1^ takes place. This redistribution evidently indicates a slight decrease of sodium content in the laser-written track similar to earlier reported for sodium borate glass and most-likely related to the thermally driven diffusion (Soret effect) of Na^+^ out of the laser-heated area [10]. However, the decrease of sodium content is expected to be less than in the earlier reported experiment in sodium borate glass [20] because there is no noticeable shift of the peaks at 772 and 965 cm^−1^. The relative intensity increase of the peaks at 460 and 1300–1600 cm^−1^ can be attributed to the growth of 3-coordinated boron content and, in particular, of [BO_2_O^−^] groups containing a non-bridging oxygen bond, which results in the decrease of the connectivity of the glass-forming network.

During the preparation of the sample for the AFM study, a high rate of laser-written tracks etching with a 0.12 M KOH solution was revealed. In this regard, a set of tracks was written according to the regime: 10 kHz, 250 nJ, 600 fs, —at various scanning speeds of 3, 30 and 300 µm/s and two orthogonal laser beam polarizations. These tracks were treated with solutions of various reagents: H_2_O (distilled), isopropyl alcohol, 0.12M KOH (aqueous), 0.12M KOH (in isopropyl alcohol). The etching rate was estimated as a ratio of the length of the etched channel to the etching time. Selectivity was calculated by dividing the sum length of etched track and etched pristine glass on the width of the etched pristine glass layer. The length of the etched track was determined by measurement dye penetration depth inside the channel (Figure S1). Figure 3 shows histograms that demonstrate etching rate (Figure 3b) and selectivity (Figure 3c) at different etching times and room temperature.

The etching rate in the series H_2_O, KOH-H_2_O, KOH-C_3_H_7_OH decreases (Figure 3b), which indicates the high affinity of laser-modified glass to water. At the same time, the decrease in the rate over time can most likely be explained by the decrease of etchant concentration inside the etched channel due to the absence of efficient mixing of the etching solution. The longer channel, the longer time required for delivery of new portions of the etching agent from the solution surrounding the sample to the track region being etched, since the etching products formed in the etched channel prevented the further penetration of the etching reagent into the depth of the channel. For the first time, we demonstrate the etching of a channel with distilled water, while this reagent achieves the highest etching rate and the highest selectivity. Adding KOH to water decreases the selectivity by about two times, with a decrease in the etching rate. Noticeably, etching practically did not occur in isopropyl alcohol, and a mixture of KOH with isopropyl alcohol showed the slowest etching rate as is presented in the graph (Figure 3a).

It can be assumed that high etching selectivity of laser-modified areas is considered to appear mainly due to the presence of hollow stretched cavities or nanopores formed in the track under laser irradiation (Figure 1) and oriented along the track in the case of the transverse orientation of the laser beam polarization plane. These cavities can significantly promote the etching process by increasing the contact surface area between the etching agent and laser-modified material and facilitating the fast percolation of the etching agent along the track. Surprisingly, the etching rate and selectivity did not depend on the writing speed as well as the polarization state of the laser beam. It is interesting to mention that independence of selectivity on the direction of laser beam polarization was also shown for fused silica in special laser writing regimes [21,22]. On the other hand, etching selectivity of the tracks written at high scanning speed and low exposure dose can be caused by the presence of defects such as non-bridging oxygens [23]. To suggest an explanation of the etching rate and selectivity in distilled water and water solution of KOH, it should be taken into account that dissolution of borate glasses proceeds through two main reactions namely hydration and hydrolysis [20]. During the hydration, a proton replaces an alkaline ion, which compensates for the excess charge of four-coordinated boron, therefore boron must reduce its coordination number to stabilize the structure. In this case, hydration of the alkaline cation is accompanied by cleavage of the B-O-B bond. During hydrolysis, water molecules attack the bridging bonds between neighboring three-coordinated boron atoms with the formation of boric acid in the solution. Since the content of three-coordinated boron increases in laser-written tracks in comparison with unmodified glass, hydrolysis is a relevant factor in the etching selectivity of the track. The highest etching speed using distilled water is caused by the joint action of both mechanisms. The introduction of potassium hydroxide in the etching solution shifts the glass dissolution reactions towards to hydrolysis mechanism, instead of both hydrolysis and hydration action in the case of water, which leads to decreasing overall track etching speed [13]. Another possible reason for the decrease of etching speed is the growth of etching solution viscosity [24] after adding KOH to water. At the same time, Al_2_O_3_ suppresses the dissolution of glass in neutral and acid medium, but not in basic one. That leads to the acceleration of the dissolution of pristine glass in basic solution and thus to the decrease of etching selectivity. Despite the fact of etching pristine alumino-contained glass, we believe, it is possible to develop approaches for modifying the chemical composition of glass with oxides which increase the chemical resistance of the glass but do not noticeably affect the laser-assisted etching selectivity.

## 4. Conclusions

In summary, the formation of nanogratings manifesting from birefringence was demonstrated in the bulk of sodium aluminoborate glass. The effect of the parameters of laser treatment on the retardance of nanogratings was determined and turned out to be similar to the effects demonstrated for binary sodium borate glass. The main differentiating feature of the formed nanogratings is the large period of the structure, 0.5–0.6 μm, as compared to nanogratings formed in silicate glasses. The possibility of selective etching of laser-written tracks with distilled water has been demonstrated for the first time. The etching rate can reach 400 μm/h at room temperature. Selectivity of the etching process varies from 100 to 400, depending on the etching solution.

## Data Availability

The data presented in this study are available on request from the corresponding author.

## Supplementary Materials

The following are available online at https://www.mdpi.com/article/10.3390/ma14195495/s1, Figure S1: optical microphotograph of etched tracks with Rodamin 6G in luminescence registration regime.
