# Supplementary material for: Hollow Channel Formation inside Sodium Aluminoborate Glass by Femtosecond Laser Writing and Distilled Water Etching
Source: Materials (Basel). 2021 Sep 23;14(19):5495. doi: 10.3390/ma14195495 (PMC8509244; doi:10.3390/ma14195495)
Supplement: Supplementary file 1 [file materials-14-05495-s001.zip › materials-1369785 -supplementary.pdf]

materials

*Supplementary Materials*

# Hollow Channel Formation inside Sodium Aluminoborate Glass by Femtosecond Laser Writing and Distilled Water Etching

**Sergey Fedotov *, Alexey Lipatiev, Tatiana Lipateva, Sergey Lotarev and Vladimir Sigaev**

Department of Chemical Technology of Glass and Glass Ceramics, Mendeleev University of Chemical Technology, 125480, Moscow, Russia; lipatievas@yandex.ru (A.L.);
t.lipateva@yandex.ru (T.L.); sergey_lot@mail.ru (S.L.); vlad.sigaev@gmail.com (V.S.)
* Correspondence: ssfedotov@muctr.ru

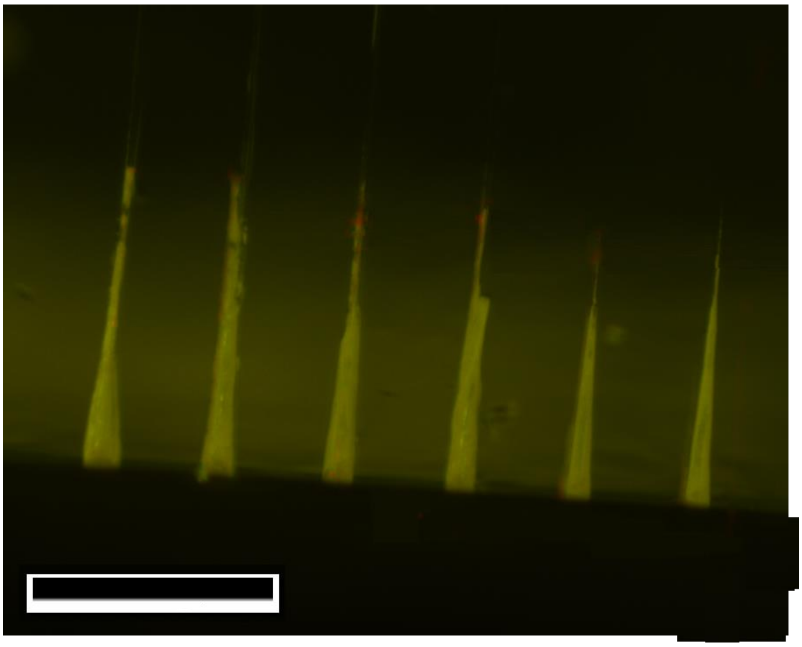

**Figure S1.** Optical microphotograph of etched tracks with Rodamin 6G in luminescence registration regime.
